# Propolis Extract: Weaving Antioxidant Power into Polymeric Composites Through Electrospinning

**DOI:** 10.3390/polym16223230

**Published:** 2024-11-20

**Authors:** Sergiana dos Passos Ramos, Leonardo Ribeiro Bernardo, Monize Bürck, Laura Ordonho Líbero, Marcelo Assis, Anna Rafaela Cavalcante Braga

**Affiliations:** 1 Department of Biosciences, Universidade Federal de São Paulo (UNIFESP), São Paulo 04021-001, Brazil; sergiana.passos@unifesp.br (S.d.P.R.); lr.bernardo@unifesp.br (L.R.B.); marcelotassis@gmail.com (M.A.); 2 Nutrition and Food Service Research Center, Universidade Federal de São Paulo (UNIFESP), São Paulo 04021-001, Brazil; monize.burck@unifesp.br; 3 Center for Development of Functional Materials (CDMF), Federal University of São Carlos (UFSCar), São Carlos 13565-905, Brazil; laura.libero@estudante.ufscar.br; 4 Department of Chemical Engineering, Universidade Federal de São Paulo (UNIFESP), São Paulo 04021-001, Brazil

**Keywords:** electrospun, nanofibers, bioactive compounds, propolis extract

## Abstract

The manufacture of composites with bioactive compounds represents a promising strategy for developing advanced materials in biomedical, food, and industrial applications. However, challenges such as stability, bioactivity retention, and controlled release hinder their effectiveness. Electrospinning emerges as a viable technique for encapsulating bioactive compounds, offering advantages such as high surface area, porosity, and gradual release, which are critical for maintaining the bioactivity of embedded compounds. Regarding bioactive composition, propolis has been highlighted as a potential source and has great potential as a biopolymer ingredient due to its antioxidant and antimicrobial properties. This study analyzed the composition and antioxidant activity of three commercial propolis extracts to select the most suitable extract for fiber composite production using zein and polyethylene oxide (PEO), both recognized as safe. The characterization of the electrospun fibers, including morphology, thermal properties, and antioxidant release, was conducted through various analytical techniques. The findings highlight the effectiveness of electrospinning for developing composite materials with bioactive compounds, paving the way for innovations in antioxidant technologies across multiple sectors.

## 1. Introduction

The encapsulation of bioactive compounds for developing new technologies represents a promising approach, particularly in the context of advanced materials for biomedical, food, and industrial applications. However, one of the main challenges lies in ensuring these bioactive compounds maintain their stability and efficacy over time [1,2]. Factors such as degradation, loss of bioactivity, or interactions with the polymeric encapsulating matrix can compromise the bioactive performance [3]. Additionally, the control of bioactive release to preserve its therapeutic or functional properties is another critical obstacle to overcome [4]. These difficulties make research in this field especially complex, requiring new strategies to protect and optimize the functionality of encapsulated compounds.

Among the various techniques for encapsulating bioactive compounds, electrospinning stands out as a promising alternative. This technique enables the production of fibers with controlled diameters, ranging from nanometers to micrometers, providing high surface area and porosity, which are ideal characteristics for efficient bioactive encapsulation [5,6]. Moreover, fibers produced via electrospinning offer the advantage of gradually releasing the encapsulated compounds, promoting controlled release, which is crucial for maintaining the bioactivity and stability of the material [7,8]. This versatility, combined with the ability to adjust the composition of the fibers and the release behavior, makes electrospinning a powerful tool in developing advanced technologies for various applications, such as biomaterials and smart packaging.

Our research group has made significant advances in encapsulating natural bioactive compounds of interest using the electrospinning technique [9,10]. Recently, we encapsulated pitanga extracts, rich in carotenoids, in fibers with diameters ranging from 4 to 6 µm, gradually releasing these carotenoids with high bioaccessibility [11]. Natural pigments, such as C-phycocyanin, anthocyanins, and carotenoids, have also been successfully encapsulated in biopolymeric nanofibers, preserving their functional and bioactive properties [12,13]. For instance, the antioxidant activity of anthocyanins extracted from jussara pulp was successfully maintained by encapsulating them in polyethylene oxide fibers, improving their thermal stability, a crucial factor for their application in food, packaging, and cosmetics [14]. These results demonstrate that electrospinning is an effective tool for encapsulating bioactive compounds and preserving their properties, expanding the possibilities for developing new high-functionality products.

One of the bioactives that has been studied since ancient times is propolis. Propolis is a resinous substance that bees collect from various plant secretions, which they use to protect and maintain stability within their hives. Beyond its role in apiculture, propolis has garnered significant attention in both traditional and modern medicine due to its rich composition of bioactive compounds and its vast biotechnological potential [15]. Due to its anti-inflammatory, antioxidant, antimicrobial, and immunostimulatory properties, propolis is considered an excellent bioactive ingredient for incorporating biomaterials, particularly into biopolymers [16]. The efficacy and compatibility of propolis are influenced by factors such as the type of extract and the biomaterial used. Propolis composition varies based on local flora; climate; and constituents like wax, pollen, and organic compounds. Its main constituents include balms and resins, fatty acids, waxes, aromatic compounds, pollen, and organic/mineral compounds [17]. Phenolic compounds, particularly flavonoids, dominate propolis and are associated with its biological activities [18]. Given its rich chemical composition and medicinal properties, propolis presents a promising opportunity as a raw material in the pharmaceuticals, cosmetics, and food industries [19].

The potential of propolis in producing electrospun nanofibers has already been reported by Du et al. [20]. The authors designed nanofibers containing propolis extract for trauma dressings that were created using silk fibroin (SF) and gelatin (GT) as the primary materials, with highly volatile formic acid as the solvent. According to the authors, propolis notably enhanced the antibacterial effectiveness against *Escherichia coli* and *Staphylococcus aureus* compared to the silk gelatin nanofiber material (SF/GT) alone. In vitro biocompatibility studies demonstrated that SF/GT-1%EP displayed favorable cytocompatibility and hemocompatibility, significantly promoting the migration of L929 cells. When applied to a mouse model with full-thickness skin defects, SF/GT-1%EP effectively accelerated wound healing. These findings suggest that the SF/GT-EP nanofiber composite exhibits excellent biocompatibility, promotes cell migration, possesses antibacterial properties, and supports healing, presenting a promising approach for treating full-thickness skin defects.

In this context, this work aimed to analyze the composition of three commercial propolis extracts in terms of their composition and antioxidant activity to select the best extract for encapsulation via electrospinning. The polymeric matrixes chosen for encapsulating this extract were zein and PEO, which are generally considered safe (GRAS) by the Food and Drug Administration (FDA). The electrospun fibers were analyzed using scanning electron microscopy (SEM), energy-dispersive X-ray spectroscopy (EDS/EDX), differential scanning calorimetry (DSC), and Fourier transform infrared spectroscopy (FTIR). The release of propolis from the fibers and its antioxidant activity were assessed using UV-Vis absorption spectroscopy and the ABTS assay, respectively. This study aims to create composites with natural bioactive and safe polymers to develop new antioxidant technologies that can be applied across different technological sectors.

## 2. Materials and Methods

### 2.1. Propolis Samples and Characterization

Three propolis (ethanolic extract) samples were purchased from the market and characterized according to their total phenolic and flavonoid compound contents. After preliminary analyses, the propolis extract from Fazenda Tamanduá^®^ (Santa Terezinha, Brazil), kindly donated by the company, was used to produce the composites.

The Folin–Ciocalteu methodology determined the total phenolic compound content, as described by Mohankumar et al. [21], with results expressed as gallic acid equivalent (GAE/g). Additionally, the total content of flavonoids present in red propolis extract was quantified according to the colorimetric method [22]. The absorbance of the solution was determined at 510 nm in a Varian^®^ spectrophotometer (Varian, Cary-50, Mulgrave, Australia). Total flavonoid contents were calculated as mg of rutin equivalent (RuE)g^−1^ using a calibration curve (R^2^ = 0.998).

### 2.2. Polymeric Solution of Propolis Extract

To prepare the propolis composites, 1% polyethylene oxide (PEO), 12% zein, and 20% propolis extract, solubilized in ethanol, were used. Among the PEO/zein tested proportions, this one resulted in fibers with a more uniform diameter. These conditions were chosen after evaluating different polymer concentrations, as described by Ramos et al. [12]. The zein and propolis extract were first incorporated into the ethanol with mechanical agitation, and then PEO was slowly added to obtain better solubilization. With the same conditions and concentrations, a solution without the active ingredient (propolis composite sample A—PCa, propolis composite sample B—PCb, and propolis composite sample C—PCc) was produced (Control) to be a comparable standard in specific tests.

### 2.3. Production of Polymer Composites by Electrospinning

The solutions produced were subjected to the electrospinning equipment (Fluidnatek LE-10, Bioinicia, Spain), where the dried samples will be deposited on an aluminum collector. They were ejected through a capillary with a diameter of 0.45 mm. The positive polarity electrode was connected to the end of the needle, and the insulation was connected to the aluminum collector. The distance between the end of the capillary and the collector was 100 mm, the applied electric potential was 20 kV, the solution flow rate was 1800 µL/h, and all experiments were conducted at room temperature and with relative humidity between 50 and 60% [12].

### 2.4. Antioxidant Activity

Samples (2 mL) were filtered, and the retentate was washed twice with an additional 10 mL of methanol in each. The filtrate was then concentrated using a rotary evaporator at temperatures around 37 °C, and the samples were filtered using 0.22 µm cellulose acetate membranes (Analítica^®^, São Paulo, Brazil) before analysis. The antioxidant activity was performed against ABTS radical [23], using Trolox as standard and data expressed as Trolox equivalent.

### 2.5. Composite Characterization

The characterization of the produced composites and their raw materials was performed based on absorption in the infrared region, analyzed utilizing a Fourier transform infrared spectrophotometer (Bruker Alpha-P, Billerica, MA, USA, in the range between 4000–400 cm^−1^) and by scanning electron microscopy (SEM) (FEG-SEM Supra 35 VP, Carl Zeiss, Oberkochen, Germany). For the FTIR analysis, the dry samples obtained were subjected to maceration with KBr and subsequently deposited in tablets and subjected to reading in the equipment. Finally, to confirm the composition of the formed structures, samples were also mapped by EDX spectroscopy (Philip XL-30 TMP FE-SEM, Bruker Alpha-P, Billerica, MA, USA, coupled to an Oxford EDS). The thermal stability of the composites was characterized by thermogravimetric analysis and differential thermal analysis (TG/DTA) (TA Instruments Q-50—Mettler-Toledo, Barueri-SP, Brazil), under a temperature range of 35 to 800 °C and an O_2_ atmosphere with a scan rate of 10 °C min^−1^. Approximately 10 mg of each dry sample was used for characterization by differential scanning calorimetry (DSC), placing them in aluminum crucibles and performing (DSC 404 C equipment controlled by TASC 424/3A—Netzsch, Germany) between 20 and 240 °C with a heating and cooling constant of 10 °C/min and a flow rate of 1 cm^3^.min^−1^. The ABTS method determined the antioxidant activity of the structures obtained from producing propolis composites according to the method previously described for the characterization of propolis extract (Section 2.2).

### 2.6. Release Evaluation

The release behavior test of the produced composite was performed by solubilizing 30 mg of the fiber in 30 mL of saline phosphate buffer (pH 7.4), maintained by stirring at 100 rpm at 37 °C for 24 h, collecting 1.5 mL aliquots (0; 2; 4; 10; 12; 18; and 24 h) at predetermined periods, which were frozen in an ultrafreezer at −40 °C, lyophilized, and resuspended in 1.5 mL of 80% ethanol and subsequently subjected to the antioxidant activity tests by the ABTS. The results calculation was based on Equation (1).

Release % = AA (t) × 100/total AA (1)

with AA (t) being the antioxidant activity (μmol of TE/g) at the time evaluated (0; 2; 4; 10; 12; 18; and 24 h) and total AA the value considered as 100% of release expressed in μmol of TE/g. Before the release of kinetic determination, an exhaustive extraction of the composite containing propolis was determined. For the PCc sample, this value was considered as total AA (100% of release) of the antioxidant activity from the incorporated propolis into the PCc for the kinetic release calculation.

## 3. Results

### 3.1. Propolis Extract Obtantion

For comparison purposes, three commercial propolis brands were evaluated to use the most promising bioactive compounds in the present study. The three extracts were assessed regarding total phenolics, total flavonoids, and antioxidant activity, and the results obtained are shown in Table 1.

The total phenolic content of the propolis used in this study, commercial sample C, was the highest compared to the results obtained by the other extracts evaluated. Nevertheless, the two brands (brand A and brand B) had similar results, while the extract used in this study reached a value approximately three times higher. Similarly, the total flavonoid content was significantly higher when compared to the other commercial brands, where this time, brands A and B had a significant difference between them, but still below the extract used, being twice as high as brand A and 1.7 times as high as brand B.

These results demonstrate a significantly higher richness of compounds, favoring the extract chosen for the present work. This difference may have been due to factors in the composition of the propolis used, which may vary depending on where it was collected and in which type of biosphere it was produced since all samples are native to Brazil, the country with the greatest biodiversity in the world and of continental proportions [16,24]; therefore, they may present different types and compositions of the natural product. Another factor, in addition to the raw material, may be the different methods of production, storage, and disposal of the products, which may interfere with the amount of phenolics and flavonoids present [17,25].

Furthermore, this behavior of total phenolics is repeated when compared to the results of the study by Tran et al. [26], in which eight different types of Australian propolis were evaluated, obtaining results for total phenolics between 1.3 and 180.5 GAE/g, with an average of 68 GAE/g per extract. In this same study, propolis of Brazilian, Moroccan, and Palestinian origin were subjected to the tests as a standard, obtaining similar results. However, the authors themselves consider that the values obtained were below what was expected and collected in the literature. As a hypothesis, they cite the difficulty of comparing total phenolic results from different studies due to different methodologies and conditions used in these tests. However, when compared to the results of Kasote et al. [27], the result is within the average obtained by the authors of 240 GAE/g of ethanolic extract of propolis from different regions of India (results between 72.05 and 454.1 GAE/g).

### 3.2. Production and Characterization of Polymer Composites by Electrospinning

**Figure 1:**
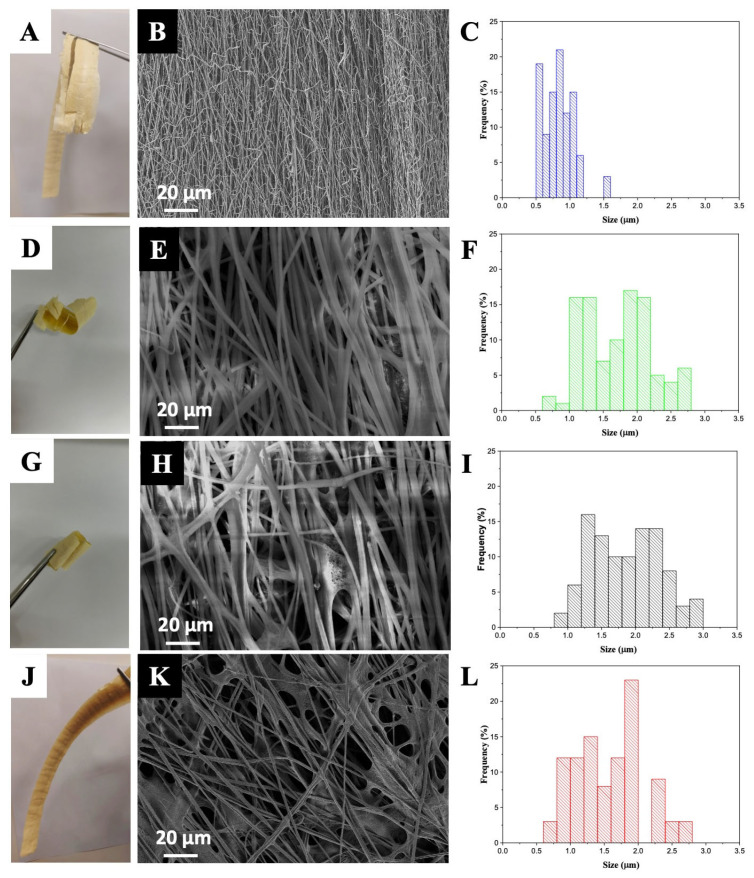
Analysis of the samples showing (**A**,**D**,**G**,**J**) photographs from samples A, B, and C (PCa, PCab, and PCc, respectively), (**B**,**E**,**H**,**K**) SEM images of the fibers, and (**C**,**F**,**I**,**L**) fiber size distribution. The control sample is represented in (**A**–**C**), the PCa sample is represented in (**D**–**F**), the PCb sample is described in (**G**–**I**), and the PCc sample is depicted in (**J**–**L**).

As previously mentioned, commercial propolis extracts A, B, and C were selected for encapsulation with PEO/zein using the electrospinning technique. Both polymers used are considered GRAS by the FDA. Therefore, they can formulate components for pharmaceutical products, food, and cosmetics (FDA UNII 16P9295IIL). In addition, studies in the literature indicate PEO and zein as excellent carriers of bioactive compounds [28,29,30]. After the production of the PC samples, a solid product with a fibrous appearance; yellow and dark coloration; and the ability to maintain the sensory characteristics of propolis, such as color and aroma, was obtained (Figure 1A,D,G,J). After this macroanalysis, the initial step in the evaluation process was to assess the integrity of the electrospun fibers. To achieve this, detailed SEM analyses were conducted to ensure proper fiber formation and structural consistency. The results of the SEM analysis for the composites; control; and propolis composite from samples A, B, and C (PCa, PCab, and PCc, respectively) are presented in Figure 1B,E,H,K. The images show that uniform; micrometric; and, in particular, mostly unidirectional fibers were obtained for the control. It is observable that the addition of propolis extract, regardless of the sample incorporated, to the zein and PEO solution significantly alters the fiber size. Analyzing the fiber diameters, the control fiber exhibited an average diameter of 0.68 ± 0.17 µm.

In contrast, the PC sample had a much larger average diameter of 1.85 ± 0.51 µm for PCa, 1.72 ± 0.44 µm for PCb, and 1.79 ± 0.58 µm for PC. This increase in fiber diameter is attributed to the interaction between the propolis extract and the polymeric components, which influences the final fiber structure, and there was no statistical difference between the analyzed samples (*p* < 0.05). The similarity in fiber diameter across the samples indicates that the bioactivity and the concentrations of phenolics and flavonoids did not alter the composite’s appearance or the size of the resulting fibers.

**Figure 2:**
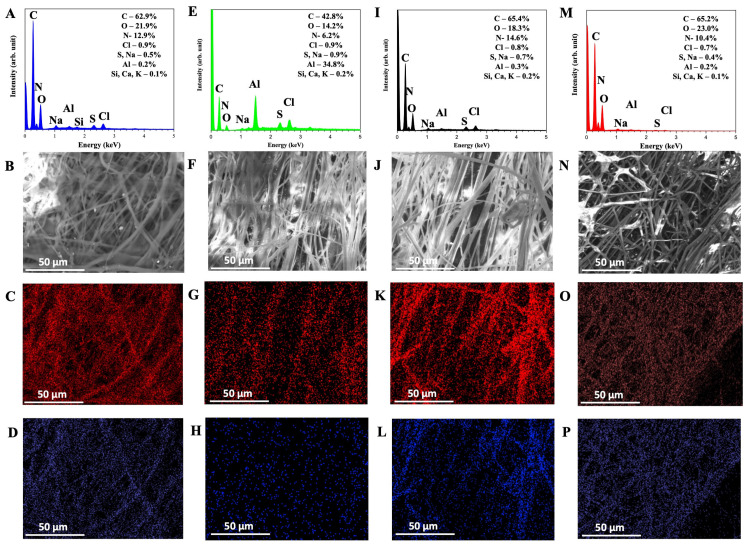
EDX spectra of the control (**A**–**D**) and PCa (**E**–**H**), PCb (**I**–**L**), and PCc (**M**–**P**) samples, re-spectively. SEM images of the control and PC samples (**C**,**F**,**J**,**N**). EDS mapping of carbon (C) (**C**,**G**,**K**,**L**) and EDS mapping of oxygen (O) (**D**,**H**,**L**,**P**) for the control and PCa, PCb, and PCc samples, respectively.

EDX analysis shows the maps’ sum spectra considering the atomic composition’s weight proportions (Figure 2) from the evaluated PCa, PCb, and PCc composites. According to the atomic composition data, the presence of carbon, oxygen, and nitrogen atoms in all samples was expected due to the organic nature of the components. The small change in concentrations between the samples also demonstrates the production of a homogeneous composite. Additionally, traces of Na, S, Cl, and Al were present in both samples. The origin of these elements in the samples evaluated may come from their presence in the raw material, zein, and/or the propolis, particularly for Na, Cl, and S, or even due to some external source. In the propolis sample, traces of Si, Ca, and K were detected at 0.1%. They were, therefore, considered irrelevant, and their presence resulted in possible external contamination. The slight change in concentrations between the samples also demonstrates the production of a homogeneous composite. A brief increase in C concentration in the samples with propolis observed was expected due to its composition, which includes phenolic compounds and resins, where the element is abundant.

The samples were mapped to complement the EDX spectra. In the mapped images of C and O atoms and the elemental mixture, it is evident that these elements are predominant in all samples, showing no significant differences in elemental distribution between them, except for PCa, which showed a lower carbon percentage than the other samples. Since propolis extract is organic in nature, it does not significantly alter the chemical contrast of the PC samples. Therefore, the compounds primarily form the fiber structures, including the propolis’s entire complex organic and bioactive content, indicating the potential for future applications with these composites.

Since they exhibited similar characteristics in both SEM and EDX analysis, the differences in terms of structural characteristics among the samples PCa, PCb, and PCc were minimal. However, they showed significant differences regarding antioxidant activity and the concentrations of phenolics and flavonoids.

**Figure 3:**
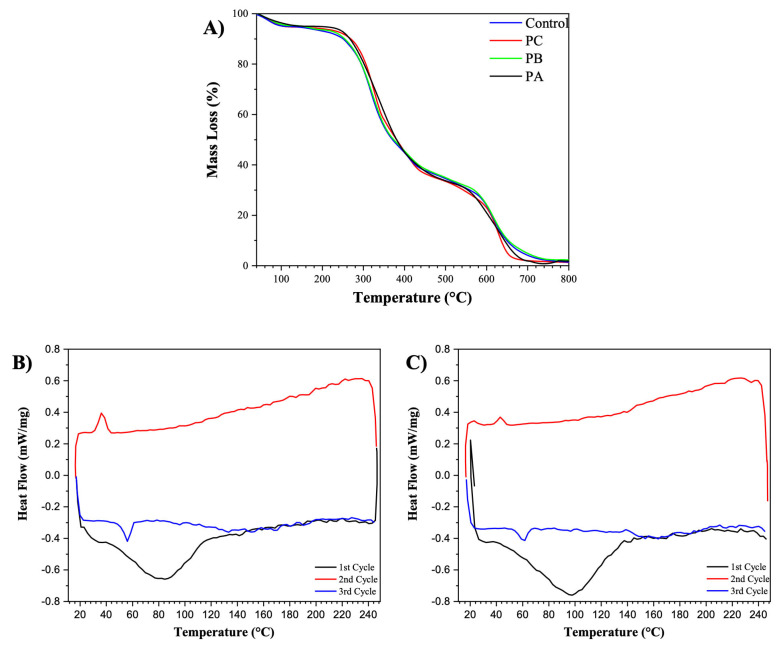
Thermal stability of the composites by TG from samples A, B, and C (**A**) and DSC analysis of control (**B**) and (**C**) PCc.

The thermal stability of the control and PC samples A, B, and C was evaluated using thermogravimetric analysis (Figure 3A). It was observed that there were no significant differences between the control sample and the PCa, PCb, and PCc samples. Thus, although the addition of propolis alters the morphology of the fibers, it does not cause any modifications in the thermal stability of either zein or PEO. Three thermal events were observed: the first (~100 °C) corresponds to the evaporation of water from the samples, the second indicates the thermal degradation of zein (~275 °C) and PEO (~320 °C), and the subsequent events are attributed to the carbonization of the residual material [9,31,32]. The DSC analysis shows that the composites presented endothermic and exothermic processes during the heating–cooling–heating cycles (Figure 3B,C). In the first heating cycle, in both samples, this was the process with the highest intensity recorded, obtaining an area value (ΔH) of −90.23 J/g and −62.28 for the control (Figure 3B) and PCc (Figure 3C), respectively, demonstrating a process with greater intensity in the composites without propolis, something that was also reflected in the initial temperature of these peaks, since the T_onset_ values obtained were higher for the control (T_onset_ = 67.7 °C and T_peak_ = 97.5 °C) when compared to the PCc (T_onset_ = 44.3 °C and T_peak_ = 85.0 °C), with one justification for this behavior being the greater thermosensitivity of propolis and its constituents.

During the second cycle, referring to cooling, both samples presented a short and low-intensity exothermic process, with ΔH = 1.35 J/g and ΔH = 4.372 J/g for the control and PCc, respectively. Thus, this demonstrates an inverse ratio of the process described previously, being a more energetic process for PCc but maintaining the pattern of occurrence before the control (T_onset_ = 31.8 °C for PCc and 39.3 °C for control). This behavior probably occurred due to the crystallization of PEO since this peak was only demonstrated in the DSC of the raw materials of this component and is typical of an amorphous polymer. According to the study by Medeiros et al. [33], the amount of PEO in zein/PEO fibers is directly proportional to the crystallization. Since 1% of the polymer is used in the manufacture of the composites, it is expected that the peak presented in this cycle will be low, which corroborates what was observed in the PEO. Finally, in the third cycle, where the samples were subjected to reheating, a similar endothermic peak was presented again at the initial temperatures for each sample, being T_onset_ = 51.5 °C for PCc and 54.4 °C for the control and of low intensity, being ΔH = −3.74 J/g and ΔH = −3.038 J/g. The similar behavior of these peaks in both samples can be related to the total degradation of the active ingredient in the first heating, in addition to demonstrating that the fibers, after being degraded, do not return to their initial state, undergoing only a final degradation, probably due to the phase change of the crystallized polymer in the previous cooling phase. Electrospun propolis/polyurethane (PU) composite nanofibers were produced by Kim et al. [34], and the obtained results show a gradual decrease in thermal stability with increasing amount of propolis in the composite.

**Figure 4:**
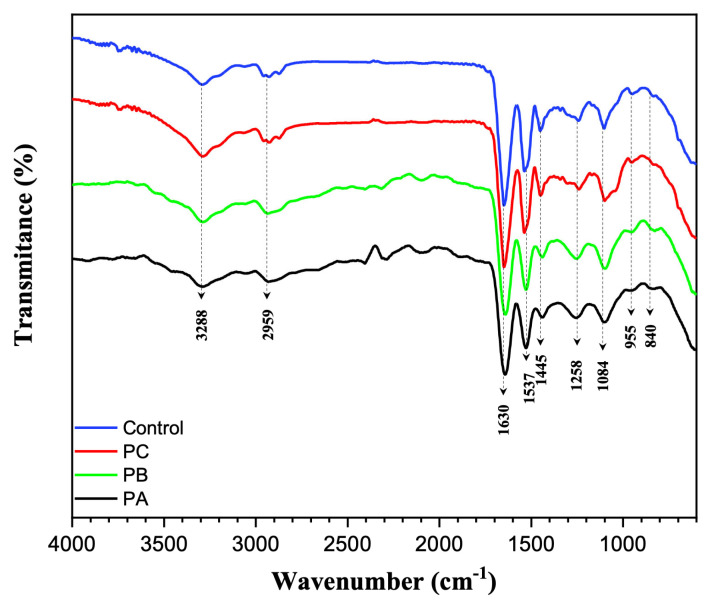
FTIR spectra from the propolis extract, control, and PCc samples.

The resulting FTIR spectra obtained from the samples of the propolis extract; control; and PC samples A, B, and C are presented in Figure 4. The typical characteristics of zein and PEO bands were similar to those of our previous reports [12,35]. In composite fibers containing propolis, there was no pronounced change in the FTIR spectrum compared to the control fibers. In the spectrum of propolis with its variable and complex composition, it presented a wide range of bands, such as the large band at 3288 cm^−1^, which may be related to the O–H stretching of flavonoids or resins, C-H of aromatics, or N–H of amino acids; the peak presented at 1630 cm^−1^, linked to C–O vibrations of esters and primary or secondary alcohols; the band at 1045 cm^−1^, which may be associated with the O–H group of various structures, such as fatty acids, carboxylic acids, and secondary alcohols; and the band at 1630 cm^−1^, which may be related to lipids and flavonoids, originating from aromatic or C=O elongations and/or from tertiary and secondary alcohol and C–H unfolding [34,36].

The behavior of the structures generated from zein and PEO, with and without the addition of propolis, presented some bands in common, as expected. The peaks corresponding to 3238 cm^−1^ may be related to the N-H elongation from zein in all samples and the O-H group of propolis compounds in the sample in which it was incorporated. The band 1537 cm^−1^ is related to the bands presented by PEO [37,38]. The presence of a triple band close to 1084 cm^−1^ is also noted, originating from the vibration of the C-O-C ether group and the sharp peak at approximately 955 cm^−1^, associated with the asymmetric stretching -C-O- of the polymer [37,39]. Zein, as it is a protein and a group of prolamins, presents in its spectrum a richness of nitrogen radicals, evidenced in the bands at 3288 and 1537 cm^−1^, which represent, respectively, the amide II, N-H stretching and can also be related to the O-H stretching; the amide I stretching and C=O vibrational stretching; and the N-H bond and C-N stretching of amides II [40,41].

### 3.3. Antioxidant Activity and Release Evaluation

All characterization analyses performed on composites containing propolis confirmed its presence in the composites. However, to ensure that the biological effects remained in the fibers, the antioxidant activity was evaluated as a response to the release test, assessing the kinetics of release of the bioactive compounds present in the composite over time. It indirectly evaluates the maintenance of the bioactive compounds in the composites containing propolis and their biological activity and the release of active compounds. Since it exhibited similar characteristics in SEM, EDX, and FTIR analysis, further release analyses were conducted solely on the PCc sample, demonstrating the most promising bioactivity.

To this end, the antioxidant activity of the ABTS method was performed on aliquots that were removed at predetermined times. The antioxidant activity was also performed with the control, and the results presented an antioxidant activity of 0.32 ± 0.05 μmol of TE/g of composite. Before the release kinetic determination, an exhaustive extraction of the composite containing propolis was determined, and for the PCc sample, the ABTS analysis resulted in a 32.66 μmol of TE/g of composite; this value was considered as 100% of the antioxidant activity from the incorporated propolis into the PCc for the kinetic release calculation. Analyzing Table 2, it is possible to observe a gradual release of the bioactive compounds in the composites, measured through their antioxidant activity. A relatively slow release was observed during the first 12 h of the release assay, reaching 27.312%. However, after 18 h, an exponential release occurred, with 61.237% of the propolis extract released. By the 24 h mark, the release had reached its peak, with approximately 95% of the extract released. The total antioxidant activity of the composite at 24 h was measured at 30.98 µmol of TE/g. This gradual release can be explained by the low solubility of zein in water (the protein has more excellent solubility in alcoholic media) [42,43]. The slow and sustained release highlights zein/PEO potential as an excipient for composites, where controlled release is essential for maximizing bioavailability and stability. The gradual release in the early stages ensures a prolonged effect. In contrast, the rapid release later provides a burst of activity when needed, maximizing the efficiency of the encapsulated propolis extract. This release is particularly advantageous in applications such as antimicrobial coatings and wound healing material, where both immediate and long-lasting effects are desirable.

The value obtained in the present study was comparable to the value obtained by Alves et al. [44] in the propolis extract in 50% ethanol (33.13 ± 1.66 µM TE/g), raising questions about the greater presence of water and the decrease in antioxidant activity, since in the cited work, a gradual reduction in antioxidant activity was presented concerning the alcohol concentration, which, the higher it was, the more potential was presented, both by ABTS and by DPPH. In addition, these results are consistent with Bonadies et al. [45], in which nanofibers were obtained in a zein and propolis composite, and a degradation test in images and release under conditions similar to those used in the present study were performed. However, with a longer time frame, they found a predominant release of the active ingredient in the period from 0 to 48 h, a fact attributed to its large contact area when compared to non-fibrous films, which allows greater water permeability and, consequently, greater release of the active ingredient. The incorporation of zein nanoparticles and Fe^3+^-encapsulated propolis extract into a pectin film was developed by Li et al. [46]. After incorporating propolis into the nanoparticles and in the film, the DPPH and ABTS scavenging capabilities were significantly enhanced, as expected due to the high phenolic content in propolis, resulting in a slight reduction in the antioxidant activity. Similar behavior was also found in the present work. Moreover, the authors affirm that a decrease in zein nanoparticles was observed, primarily due to the encapsulation of propolis by the zein nanoparticles, which limits the release of propolis extract. Conversely, the introduction of Fe^3+^ led to a slight enhancement in the composite film’s DPPH and ABTS scavenging capacities, mainly attributed to the increased electron-withdrawing capability of propolis extract when Fe^3+^ is present.

## 4. Conclusions

The findings of this study confirm that it is feasible to produce propolis-based composites with macroscopic film characteristics and microscopic fiber structures using electrospinning of zein and PEO polymer solutions. Characterization tests revealed that propolis was successfully incorporated into the composite and, more importantly, retained its biological activity after fiber formation. Since characteristics in SEM, EDX, TGA, and FTIR analysis were exhibited by all the composite samples, the differences in terms of structural characteristics among the samples PCa, PCb, and PCc were minimal. However, they showed significant differences regarding antioxidant activity and the concentrations of phenolics and flavonoids. The release tests, performed only on the PCc sample, as it demonstrated the most promising bioactivity, showed a significant release of the encapsulated content between 18 and 24 h, indicating that the components enable effective and controlled release. While propolis extract demonstrated its high applicability and benefits in product incorporation, its thermal instability remains challenging. Although thermogravimetric analysis suggests some improvement in thermal stability with the electrospinning process, further refinement is needed to address this issue comprehensively. The electrospinning technique for producing propolis composites using PEO and zein is promising, with potential applications in pharmaceuticals, cosmetics, and food industries. These results highlight the viability of these composites for applications that require the gradual release of bioactive compounds, encouraging further exploration of their functional uses in diverse industries.

## Figures and Tables

**Table 1:** Total phenolic (GAE/g), total flavonoid (RuE/g), and antioxidant activity (µM TE/g) values of the propolis extract samples used and commercial standards.

Sample	Total Phenolic (GAE/g)	Total Flavonoid (RuE/g)	ABTS (µM TE/g)
Commercial Propolis A	65.6 ^b^ ± 2.44	362.14 ^c^ ± 6.88	355.30 ^b^ ± 14.04
Commercial Propolis B	67.8 ^b^ ± 1.29	444.47 ^b^ ± 10.44	349.82 ^b^ ± 15.10
Commercial Propolis C	229.8 ^a^ ± 6.39	786.11 ^a^ ± 18.39	1.188.89 ^a^ ± 75.02

Values followed by different superscript letters in each column are significantly different (*p* ≤ 0.05), determined by one-way ANOVA and Tukey’s post hoc test (Jamovi 2.6.2).

**Table 2:** The release behavior test of the propolis composites over time.

Time (h)	ABTS (μmol of TE/g)	Release %
0	0.0 ± 0.0	0.00 ± 0.00
2	5.21 ± 1.29	15.95 ± 0.12
4	5.39 ± 0.39	16.50 ± 0.23
10	6.77 ± 0.39	20.73 ± 0.19
12	8.92 ± 0.39	27.31 ± 0.43
18	20.00 ± 0.39	61.24 ± 1.20
24	30.98 ± 0.39	94.86 ± 0.55

## Data Availability

The raw data supporting the conclusions of this article will be made available by the authors upon request.
